# Additions to Phaeosphaeriaceae (Pleosporales): *Elongaticollum* gen. nov., *Ophiosphaerella taiwanensis* sp. nov., *Phaeosphaeriopsis beaucarneae* sp. nov. and a new host record of *Neosetophoma poaceicola* from Musaceae

**DOI:** 10.3897/mycokeys.70.53674

**Published:** 2020-07-27

**Authors:** Danushka S. Tennakoon, Kasun M. Thambugala, Dhanushka N. Wanasinghe, Eleni Gentekaki, Itthayakorn Promputtha, Chang-Hsin Kuo, Kevin D. Hyde

**Affiliations:** 1 Department of Plant Medicine, National Chiayi University, 300 Syuefu Road, Chiayi City 60004, Taiwan; 2 School of Science, Mae Fah Luang University, Chiang Rai 57100, Thailand; 3 Center of Excellence in Fungal Research, Mae Fah Luang University, Chiang Rai, 57100, Thailand; 4 Genetics and Molecular Biology Unit, Faculty of Applied Sciences, University of Sri Jayewardenepura, Gangodawila, Nugegoda, Sri Lanka; 5 CAS Key Laboratory for Plant Biodiversity and Biogeography of East Asia (KLPB), Kunming Institute of Botany, Chinese Academy of Science, Kunming 650201, Yunnan, China; 6 Department of Biology, Faculty of Science, Chiang Mai University, Chiang Mai 50200, Thailand; 7 Environmental Science Research Center, Faculty of Science, Chiang Mai University, Chiang Mai, 50200, Thailand; 8 Institute of Plant Health, Zhongkai University of Agriculture and Engineering, Haizhu District, Guangzhou 510225, China

**Keywords:** Asparagaceae, Dothideomycetes, leaf litter, new taxa, Zingiberaceae

## Abstract

A novel ascomycetous genus, *Elongaticollum*, occurring on leaf litter of *Hedychium coronarium* (Zingiberaceae) in Taiwan, is described and illustrated. *Elongaticollum* is characterized by dark brown to black, superficial, obpyriform, pycnidial conidiomata with a distinct elongate neck, and oval to oblong, hyaline, aseptate conidia. Phylogenetic analyses (maximum likelihood, maximum parsimony and Bayesian) of combined ITS, LSU, SSU and *tef1*-α sequence data revealed *Elongaticollum* as a distinct genus within the family Phaeosphaeriaceae with high statistical support. In addition, *Ophiosphaerella taiwanensis* and *Phaeosphaeriopsis beaucarneae* are described as new species from dead leaves of *Agave tequilana* and *Beaucarnea recurvata* (Asparagaceae), respectively. *Neosetophoma poaceicola* is reported as a new host record from dead leaves of *Musa acuminata* (Musaceae). Newly described taxa are compared with other similar species and comprehensive descriptions and micrographs are provided.

## Introduction

Plant litter is considered as one of the main contributors to net above-ground primary productivity of terrestrial ecosystems (Swift et al. 1979; Berg and McClaugherty 2008; Krishna and Mohan 2017). Since plant litter is returned back to the soil, it represents a major source of organic carbon in forest soils (Berg 2003). Plant litter can be defined as a collection of fallen leaves, twigs, seeds and other woody debris that accumulate on the ground as a natural part of the forest ecosystem (Johnson and Catley 2002; Berg and McClaugherty 2008). In particular, leaf litter is the main source of organic matter and nutrients of the soil, compared to other litter types (Robertson and Paul 1999; Berg and McClaugherty 2008; Krishna and Mohan 2017). Leaf litter decomposition is a key process contributing to biogeochemical cycles in any forest ecosystem. Microorganisms are the primary agents in this process (Purahong et al. 2016; Mlambo et al. 2019). Fungi are considered as the “key players” in leaf litter decomposition, because of their ability to produce a wide range of extracellular enzymes (Pointing et al. 2005; Berg and McClaugherty 2008; Bani et al. 2018). Many researchers have been carrying out studies of fungal species inhabiting leaf litter and have described numerous new species in Dothideomycetes (Hyde et al. 2019; Phookamsak et al. 2019; Tennakoon et al. 2019).

The family Phaeosphaeriaceae is considered to be one of the most species-rich families in Dothideomycetes and includes species that inhabit a wide range of ecosystems (i. e., marine, terrestrial, and mangroves) (Phookamsak et al. 2014, 2017; Bakhshi et al. 2019; Jones et al. 2019; Luo et al. 2019; Tennakoon et al. 2019). Phaeosphaeriaceae was established by Barr (1979), who designated *Phaeosphaeria* I. Miyake as the generic type of the family. Phaeosphaeriaceae species have immersed to superficial, globose to subglobose ascomata, short papilla, bitunicate asci and hyaline to pigmented, fusiform to ellipsoidal, filiform, or muriform ascospores (Bakhshi et al. 2019; Chaiwan et al. 2019; Maharachchikumbura et al. 2019; Yang et al. 2019). Members of Phaeosphaeriaceae are cosmopolitan, since they exhibit diverse lifestyles as saprobes, endophytes and pathogens of economically important plants (Barr 1992; Phookamsak et al. 2014, 2017; Yang et al. 2016; Hyde et al. 2020; Mapook et al. 2020). Apart from being cosmopolitan in nature, it appears that this family is phylogenetically highly diverse. Thus, recent studies have revealed a large number of new genera in this family. For instance, in the space of two years, eleven genera have been introduced, *viz. Bhagirathimyces* S.M. Singh & S.K. Singh (Hyde et al. 2020), *Hydeomyces* Maharachchikumbura et al. (Maharachchikumbura et al. 2019), *Hydeopsis* J.F. Zhang et al. (Zhang et al. 2019), *Neostagonosporella* C.L. Yang, et al. (Yang et al. 2019), *Parastagonosporella* M. Bakhshi, Arzanlou & Crous (Bakhshi et al. 2019), *Pseudoophiosphaerella* J.F. Zhang et al. (Zhang et al. 2019), *Murichromolaenicola* Mapook & K.D. Hyde (Mapook et al. 2020), *Neoophiobolus* Mapook & K.D. Hyde (Mapook et al. 2020), *Paraleptospora* Mapook & K.D. Hyde (Mapook et al. 2020), *Pseudostaurosphaeria* Mapook & K.D. Hyde (Mapook et al. 2020) and *Vittaliana* Devadatha et al. (Devadatha et al. 2019). Currently, more than 70 genera are accommodated in this family (Wanasinghe et al. 2018; Bakhshi et al. 2019; Maharachchikumbura et al. 2019; Phookamsak et al. 2019; Hongsanan et al. 2020; Hyde et al. 2020).

We are investigating the diversity of microfungi on leaf litter in the tropics with the aim of clarifying their taxonomy based on morphology coupled with multi-gene phylogeny. As a part of this study, we have collected and isolated four taxa from Taiwan, which belong to the family Phaeosphaeriaceae. We present herein comprehensive morphological descriptions and an in-depth phylogenetic investigation of the newly introduced species.

## Materials and methods

### Sample collection, morphological studies and isolation

Decaying leaf litter samples of *Agave tequilana* F.A.C. Weber (Asparagaceae), *Beaucarnea recurvata* Lem. (Asparagaceae), *Hedychium coronarium* J.Koenig (Zingiberaceae), and *Musa acuminata* Colla (Musaceae) were collected from Dahu Forest Area in Chiayi, Taiwan and taken to the laboratory in Zip lock plastic bags. Specimens were examined with a LEICA EZ4 stereomicroscope. Micro-morphological characters were determined using AXIOSKOP 2 PLUS compound microscope and images were captured with a Zeiss AXIOCAM 506 COLOR digital camera. Observations and photomicrographs were made from materials mounted in water. Permanent slides were preserved in lactoglycerol, sealed by applying nail-polish around the margins of cover slip. All measurements were made with ZEN2 (blue edition) and images used for figures were processed with Adobe Photoshop CS3 Extended version 10.0 software (Adobe Systems, USA).

Single ascospore and conidial isolation was carried out following the method described in Phookamsak et al. (2014). The single germinated spore was picked up and transferred to potato dextrose agar (PDA) and incubated at 25 °C in natural light. Subsequent sub-culturing was done carefully to obtain pure culture and ensure absence of contaminants. Culture characteristics were observed after three weeks. Colonies were photographed and colonial characters were noted and described. Type specimens of new taxa were deposited at the herbarium of Mae Fah Luang University (MFLU) and National Chiayi University Herbarium (NCYU). Living cultures were deposited in Mae Fah Luang University Culture Collection (MFLUCC) and National Chiayi University Culture Collection (NCYUCC). Faces of Fungi and Index Fungorum numbers were provided as in Jayasiri et al. (2015) and Index Fungorum (2020).

### DNA extraction and PCR amplification

Total genomic DNA was extracted from scraped fresh fungal mycelium using the DNA extraction kit E.Z.N.A Fungal DNA Mini Kit (D3390-02, Omega Bio-Tek) following the manufacturer’s protocol. The DNA product was kept at 4 °C for DNA amplification and maintained at -20 °C for long term storage. DNA was amplified by polymerase chain reaction (PCR) for four genes, the large subunit (28S, LSU), small subunit (18S, SSU), internal transcribed spacers including the 5.8s rDNA (ITS1-5.8S-ITS2) and translation elongation factor 1 alpha (tef1-α). The partial LSU gene was amplified by using the primer combination LR0R and LR5 (Vilgalys and Hester 1990; Rehner and Samuels 1994); partial SSU was amplified with NS1 and NS4 (White et al. 1990), nuclear ITS was amplified with primers ITS5 and ITS4 (White et al. 1990), and *tef1*-α gene was amplified using the primers EF1-983F and EF1-2218R (Rehner et al. 2001). Amplification reactions were performed in 25 µl of total reaction that contained 9.5 µl of sterilized water, 12.5 µl of 2×Power Taq PCR MasterMix (Tri-I Biotech, Taipei, Taiwan), 1 μl of each forward and reverse primers and 1 μl of DNA template. The PCR thermal cycle program of ITS, LSU, SSU and *tef1*-α gene was processed initially at 94 °C for 3 minutes, followed by 35 cycles of denaturation at 94 °C for 30 seconds, annealing at 55 °C for 50 seconds, elongation at 72 °C for 1 minute and a final extension at 72 °C for 10 minutes and a holding temperature of 4 °C. The PCR products were analyzed by 1.5% agarose gels containing the Safeview DNA stain (GeneMark, Taipei, Taiwan) to confirm their expected molecular weight. PCR products were purified and sequenced with primers mentioned above by Tri-I Biotech, Taipei, Taiwan. Nucleotide sequences were deposited in GenBank (Table 1).

### Phylogenetic analysis

Phylogenetic analyses were performed using a combined LSU, SSU, ITS and *tef1*-α sequence dataset. Newly generated sequence data were initially subjected to blast search in NCBI to obtain the closest matches in GenBank. Sequences generated from this study were analyzed with related taxa in the family Phaeosphaeriaceae, which were obtained from GenBank and from recently published data (Bakhshi et al. 2019; Hyde et al. 2019; Maharachchikumbura et al. 2019; Yang et al. 2019; Mapook et al. 2020) (Table 1). The combined dataset consisted of 168 sequences including our newly generated sequences. Multiple alignments were automatically made with MAFFT v. 7 at the web server (http://mafft.cbrc.jp/alignment/server), using default settings (Katoh and Standley 2013). The alignment was refined manually with BioEdit v. 7.0.5.2 (Hall 1999), where necessary.

Evolutionary models for phylogenetic analyses were selected independently for each locus using MrModeltest v. 3.7 (Posada and Crandall 1998) under the Akaike Information Criterion (AIC). Phylogenetic trees were obtained from Randomized Accelerated Maximum Likelihood (RAxML), maximum parsimony analysis (MP) and Bayesian inference analyses (BI). ML trees were generated using the RAxML-HPC2 on XSEDE (8.2.8) (Stamatakis et al. 2008; Stamatakis 2014) in the CIPRES Science Gateway platform (Miller et al. 2010) using GTR+I+G model of evolution. The MP analysis was performed using PAUP (Phylogenetic Analysis Using Parsimony) version 4.0b10 (Swofford 2002), with parameters as described in Tennakoon et al. (2019). Descriptive tree statistics for parsimony, such as Tree Length (TL), Consistency Index (CI), Retention Index (RI), Relative Consistency Index (RC) and Homoplasy Index (HI) were calculated.

The BI analysis was conducted with MrBayes v. 3.1.2 (Huelsenbeck and Ronquist 2001) to evaluate posterior probabilities (PP) (Rannala and Yang 1996; Zhaxybayeva and Gogarten 2002) by Markov Chain Monte Carlo sampling (MCMC). Six MCMC chains were run simultaneously, starting from random trees for 3,000,000 generations. Trees were sampled every 100^th^ generation for a total of 30,000 trees. The first 6,000 trees were discarded as the burn-in phase of each analysis. Posterior probabilities (Rannala and Yang 1996) were determined from a majority-rule consensus tree generated with the remaining 24,000 trees. Phylograms were visualized with FigTree v1.4.0 (Rambaut 2012) and annotated in Microsoft Power Point (2010). Sequences of the new strains generated in this study are deposited in GenBank. The final alignment and trees were deposited in TreeBASE, submission ID: 26088.

**Table 1.** GenBank and culture collection accession numbers of species included in the present phylogenetic study. Newly generated sequences are shown in bold.

Species	Strain/Voucher no.	GenBank accession no.			
		LSU	SSU	ITS	*tef1*–α
*Acericola italica*	MFLUCC 13-0609	MF167429	MF167430	MF167428	–
*Allophaeosphaeria muriformia*	MFLUCC 13-0277	KX910089	KX950400	KX926415	–
*Alloneottiosporina thailandica*	MFLUCC 15-0576	–	–	–	–
*Amarenographium ammophilicola*	MFLU 17-2571	MN017847	MN017913	MN047087	MN077065
*Amarenomyces dactylidis*	KUMCC 18-0154	MK356345	MK356359	MK356371	–
*Arezzomyces cytisi*	MFLUCC 15-0649	KT306950	KT306954	KT306947	–
*Banksiophoma australiensis*	CBS 142163	KY979794	–	KY979739	KY979889
*Bhagirathimyc es himalayensis*	AMH 10127	MK836020	MN121697	MK836021	–
*Bhatiellae rosae*	MFLUCC 17-0664	MG828989	MG829101	MG828873	–
*Brunneomurispora lonicerae*	KUMCC 18-0157	MK356346	MK356360	MK356373	MK359065
*Camarosporioides phragmitis*	MFLUCC 13-0365	KX572345	KX572350	KX572340	KX572354
*Chaetosphaeronema achilleae*	MFLUCC 16-0476	KX765266	–	KX765265	–
*C. hispidulum*	CBS 216.75	KF251652	EU754045	KF251148	KF253108
*Dactylidina shoemaker*	MFLUCC 14-0963	MG829003	MG829114	MG828887	MG829200
*Dematiopleospora cirsii*	MFLUCC 13-0615	KX274250	–	KX274243	KX284708
*D. mariae*	MFLUCC 15-0612	KJ749653	KJ749652	KX274244	KJ749655
*Didymocyrtis xanthomendozae*	CBS 129666	–	–	KP170651	KP170677
*Diederichomyces ficuzzae*	CBS 128019	JQ238616	–	KP170647	KP170673
*Dlhawksworthia clematidicola*	MFLUCC 17-0693	MG829038	MG829144	MG828929	–
*D. lonicera*	MFLUCC 14-0955	MG829012	MG829121	MG828902	MG829203
*Edenia gomezpompae*	JLCC 34533	–	–	KC193601	–
	LVPEI 3225	–	–	KU578033	–
***Elongaticollum hedychii***	**MFLUCC 18-1638**	**MT321810**	**MT321803**	**MT321796**	**MT328753**
***E. hedychii***	**MFLUCC 17-2653**	**MT321811**	**MT321804**	**MT321797**	**MT328754**
	**NCYUCC 19-0286**	**MT321812**	**MT321805**	**MT321798**	**MT328755**
*Embarria clematidis*	MFLUCC 14-0652	KT306953	KT306956	KT306949	–
	MFLUCC 14-0976	MG828987	MG829099	MG828871	MG829194
*Equiseticola fusispora*	MFLUCC 14-0522	KU987669	KU987670	KU987668	MG520895
*Galiicola baoshanensis*	HKAS 102234	MK356348	MK356362	MK356374	MK359066
*G. pseudophaeosphaeria*	MFLU 14-0524	–	–	–	MG520896
*Hydeomyces desertipleosporoides*	SQUCC 15259	MK290839	MK290843	MK290841	MK290848
	SQUCC 15260	MK290840	MK290844	MK290842	MK290849
*Hydeopsis verrucispora*	SD 2016-5	MK522498	MK522504	MK522508	MK523388
*Italica achilleae*	MFLUCC 14-0955	MG829012	MG829121	MG828902	MG829203
*I. luzulae*	MFLUCC 14-0932	KT306951	–	–	–
*Jeremyomyces labinae*	CBS 144617	MK442529	–	MK442589	MK442695
*Juncaceicola italica*	MFLUCC 13-0750	–	–	KX500110	MG520897
*J. luzulae*	MFLUCC 13-0780	KX449530	KX449531	KX449529	–
*Kwanghwaensis miscanthi*	FU31017	MK503823	MK503829	MK503817	MT009126
*Leptosphaeria doliolum*	CBS 505.75	GU301827	GU296159	JF740205	GU349069
*Leptospora rubella*	CPC 11006	DQ195792	DQ195803	DQ195780	–
*L. thailandica*	MFLUCC 16-0385	KX655549	KX655554	KX655559	KX655564
*Longispora clematidis*	MFLU 15–1277				
*Loratospora aestuarii*	CBS 117592	–	–	MH863024	–
*Mauginiella scaettae*	CBS 239.58	MH869303	–	MH857770	–
*Melnikia anthoxanthii*	MFLUCC 14-1011	KU848204	KU848205	–	–
*Murichromolaenicola chiangraiensis*	MFLUCC 17-1488	MN994559	MN994605	MN994582	MN998163
*M. chromolaenae*	MFLUCC 17-1489	MN994560	MN994606	MN994583	MN998164
*Muriphaeosphaeria galatellae*	MFLUCC 14-0614	KT438329	KT438331	KT438333	MG520900
	MFLUCC 15-0769	KT438330	KT438332	–	–
*Neoophiobolus chromolaenae*	MFLUCC 17-1467	MN994562	MN994606	MN994583	MN998164
*N. chromolaenae*	MFLUCC 17-1449	MN994561	MN994607	MN994584	MN998165
*Neosetophoma* sp.	MFLUCC 17-0844	MG829035	MG829141	MG828926	MG829219
*N. aseptata*	CBS 145363	MK540024	–	MK539953	–
*N. camporesii*	MFLUCC 15-0682	KU302778	–	KU302779	–
*N. clematidis*	MFLUCC 13-0734	KP684153	KP684154	KP744450	–
*N. garethjonesii*	MFLUCC 14-0528	–	KY501126	–	KY514402
*N. guiyangensis*	GZ13	MH018132	MH018136	MH018134	MH051889
*N. italica*	MFLU 14-0809	KP711361	KP711366	KP711356	–
*N. lonicerae*	KUMCC 18-0155	MK356349	MK356363	MK356375	MK359067
*N. lunariae*	CPC 26671	KX306789	–	KX306763	–
*N. miscanthi*	MFLU 18-2675	MK503826	MK503832	MK503820	–
*N. phragmitis*	CBS 145364	MK540025	–	MK539954	MK540148
*N. poaceicola*	MFLUCC 16-0886	KY550382	KY550383	KY568986	–
	**MFLUCC 18-1632**	**MT321809**	**MT321802**	**MT321795**	–
*N. rosae*	MFLUCC 17-0844	MG829035	MG829141	MG828926	MG829219
*N. rosaena*	MFLUCC 17-0768	MG829037	MG829143	MG828928	–
*N. rosarum*	MFLU 17-0308	MG829036	MG829142	MG828927	–
*N. salicis*	MFLU 17-0118	MK608026	–	MK608025	–
*N. samarorum*	CBS 138.96	KF251664	GQ387517	MH862569	KF253119
*N. sambuci*	CBS 145365	MK540026	–	MK539955	MK540149
*N. shoemakeri*	MFLU 16-1606	MG602199	MG602201	MG602203	MG844352
	MFLUCC 17-0780	MG844348	MG844350	MG844346	MG844352
*N. tienshanensis*	MFLUCC 17-0844	MG829035	MG829141	MG828926	MG829219
*N. xingrensis*	GZAAS18 0100	MH018133	–	MH018135	–
*Neosphaerellopsis thailandica*	CPC 21659	KP170721	–	KP170652	KP170678
*Neostagonospora caricis*	CBS 135092	KF251667	–	KF251163	–
*N. phragmitis*	MFLUCC 16-0493	KX910090	KX950401	KX926416	MG520902
*Neostagonosporella sichuanensis*	MFLUCC 18-1228	–	–	–	MK313854
	MFLUCC 18-1231	–	–	–	MK313851
*Neosulcatispora agaves*	CPC 26407	KT950867	–	KT950853	–
*Nodulosphaeria multiseptata*	MFLUCC 15-0078	KY496728	–	KY496748	–
*N. scabiosae*	MFLUCC 14-1111	KU708846	KU708842	KU708850	KU708854
*Ophiobolopsis italica*	MFLUCC 17-1791	MG520959	MG520977	MG520939	MG520903
*Ophiobolus disseminans*	MFLUCC 17-1787	MG520961	MG520980	MG520941	MG520906
*O. rossicus*	MFLU 17-1639	MG520964	MG520983	MG520944	MG520909
*Ophiosimulans tanaceti*	MFLUCC 14-0525	KU738891	KU738892	KU738890	MG520910
*Ophiosphaerella agrostidis*	MFLUCC 11-0152	KM434281	KM434290	KM434271	KM434299
	MFLUCC 12-0007	KM434282	KM434291	KM434272	KM434300
	MFLUCC 16-0895	MF197563	MF351604	MF351996	–
	IGM35	MF197563	MF351604	–	–
	MFLUCC 11-0152	KM434281	KM434290	KM434271	KM434299
*O. aquatica*	MFLUCC 14-0033	KX767089	KX767090	KX767088	MG520911
	MFLUCC 14-0033	KX767089	KX767090	KX767088	MG520911
*O. herpotricha*	k28	–	–	KP690992	KP691016
	KS29	–	–	KP690986	KP691015
*O. korrae*	ATCC 56289	–	–	KC848509	KC848515
*O. narmari*	ATCC 64688	–	–	KC848510	KC848516
	ATCC 201719	–	–	KC848508	KC848514
***O. taiwanensis***	**MFLU 18-2534**	**MT321815**	**MT321808**	**MT321801**	**MT328758**
*O. taiwanica*	NTUCC 17-024	MN082419	–	MN082417	–
	NTUCC 17-025	MN082420	–	MN082418	–
*Paraleptosphaeria dryadis*	CBS 643.86	GU301828	KC584632	JF740213	GU349009
*Paraleptospora chromolaenae*	MFLUCC 17-1481	MN994563	MN994609	MN994587	MN998167
*P. chromolaenicola*	MFLUCC 17-1450	MN994564	MN994610	MN994588	MN998168
*Paraophiobolus arundinis*	MFLUCC 17-1789	MG520965	MG520984	MG520945	MG520912
*P. plantaginis*	MFLUCC 17-0245	KY815010	KY815012	KY797641	MG520913
*Paraloratospora camporesii*	MFLU 18-0915	MN756637	MN756635	MN756639	–
*Paraphoma chrysanthemicola*	CBS 522.66	KF251670	GQ387521	KF251166	KF253124
*P. radicina*	CBS 111.79	KF251676	EU754092	KF251172	KF253130
*Parastagonospora dactylidis*	MFLUCC 13-0375	KU058722	–	KU058712	–
*Parastagonosporella fallopiae*	CBS 135981	MH460545	–	MH460543	MH460549
*P. fallopiae*	CCTU 1151-1	MH460546	–	MH460544	MH460550
*Phaeopoacea muriformis*	MFLUCC 17-0372	MF611638	MF611639	MF611637	–
*P. festucae*	MFLUCC 17-0056	KY824767	KY824769	KY824766	–
*Phaeoseptoriella zeae*	CBS 144614	MK442547	–	MK442611	MK442702
*Phaeosphaeria musae*	MFLUCC 11-0133	KM434277	KM434287	KM434267	KM434296
*P. oryzae*	CBS 110110	KF251689	GQ387530	KF251186	–
*P. papayae*	CBS 135416	–	–	MH866082	–
*Phaeosphaeriopsis agapanthi*	CPC 26303	KX228311	–	KX228260	–
*P. agavacearum*	CPC 29122	KY173520	–	KY173430	–
*P. agavensis*	CBS 102206	KY090669	KY090693	KY090635	–
*P. aloes*	CBS 145367	MK540030	–	MK539959	MK540153
*P. aloicola*	CBS 145368	MK540031	–	MK539960	MK540154
*P. amblyospora*	CBS 110131	–	–	MH862851	–
***P. beaucarneae***	**MFLU 18-2586**	**MT321813**	**MT321806**	**MT321799**	**MT328756**
	**MFLU 18-2587**	**MT321814**	**MT321807**	**MT321800**	**MT328757**
*P. dracaenicola*	MFLUCC 11-0157	KM434283	KM434292	KM434273	KM434301
*P. glaucopunctata*	MFLUCC 13-0265	KJ522477	KJ522481	KJ522473	MG520918
*P. grevilleae*	CBS 145369	MK540032	–	MK539961	MK540155
*P. nolinae*	CBS 102205	KY090667	KY090694	KY090637	–
*P. obtusispora*	CBS 246.64	JX681119	–	KY090644	–
*P. omaniana*	SQUCC:14333	MT075849	–	MT075840	–
*P. phacidiomorpha*	CBS 198.35	AF275496	AF275515	FJ462742	–
*P. pseudoagavacearum*	CBS 145370	MK540033	–	MK539962	–
	MFLU 17-1800A	MN750592	MN750607	MN750613	MN756837
*P. triseptata*	MFLUCC 13-0271	KJ522479	KJ522484	KJ522475	MG520919
*P. yuccae*	MFLUCC 16-0558	KY554481	KY554480	KY554482	MG520920
*Piniphoma wesendahlina*	CBS 145032	MK442551	–	MK442615	MK442706
*Populocrescentia ammophilae*	MFLUCC 17-0665	MG829059	MG829164	MG828949	MG829231
*P. rosacea*	MFLU 17-0128	MG829060	MG829165	–	MG829232
*Pseudoophiobolus achilleae*	MFLU 17-0925	MG520966	–	MG520946	–
*P. galii*	MFLUCC 17-2257	MG520967	MG520989	MG520947	MG520926
*Pseudoophiosphaerella huishuiensis*	HS13	MK522499	MK522505	MK522509	MK523389
*Pseudophaeosphaeria rubi*	MFLUCC 14-0259	KX765299	KX765300	KX765298	MG520934
*Pseudostaurosphaeria chromolaena*	MFLUCC 17-1490	MN994570	MN994616	MN994593	MN998174
*P. chromolaenicola*	MFLUCC 17-1491	MN994571	MN994617	MN994594	MN998175
*Poaceicola arundinis*	MFLU 16-0158	MG829057	MG829162	MG828947	MG829229
*P. bromi*	MFLUCC 13-0739	KU058727	–	KU058717	–
*Sclerostagonospora rosicola*	MFLUCC 15-0129	MG829068	MG829172	MG828957	MG829237
*Scolicosporium minkeviciusii*	MFLUCC 12-0089	KF366382	KF366383	–	–
*Septoriella phragmitis*	CPC 24118	KR873279	–	KR873251	–
*S. pseudophragmitis*	CBS 145417	–	–	MK560161	MK559452
*Setomelanomma holmii*	CBS 110217	GU301871	GU296196	KT389542	GU349028
*Setophoma antiqua*	LC6594	MK511947	–	MK511909	MK525070
*S. chromolaenae*	CBS 135105	KF251747	–	KF251244	KF253195
*S. endophytica*	LC3163	MK511956	–	MK511931	MK525092
*S. longinqua*	LC6593	MK511946	–	MK511908	MK525069
*S. pseudosacchari*	CBS 145373	MK540039	–	MK539969	
*S. sacchari*	MFLUCC 11-0154	KJ476146	KJ476148	KJ476144	KJ461319
	MFLUCC 12-0241	KJ476147	KJ476149	KJ476145	KJ461318
*S. terrestris*	CBS 335.29	KF251749	GQ387526	KF251246	KF253196
*S. vernoniae*	CBS 137988	KJ869198	–	KJ869141	MK540162
*S. yingyisheniae*	LC12696	MK511950	–	MK511914	MK525075
*S. yunnanensis*	LC6532	MK511945	–	MK511907	MK525068
*Stagonospora foliicola*	CBS 110111	KF251759	EU754118	KF251256	KF253206
*Sulcispora* sp.	MFLUCC 14-0995	KP271444	KP271445	KP271443	MH665366
*Sulcispora pleurospora*	CBS 460.84	–	–	AF439498	–
*Tintelnotia destructans*	CBS 127737	KY090664	KY090698	KY090652	–
*T. opuntiae*	CBS 376.91	GU238123	GU238226	KY090651	–
*Vagicola vagans*	CBS 604.86	KU058727	–	KF251193	KF253149
*Vittaliana mangrovei*	NFCCI 4251	MG767312	MG767313	MG767311	MG767314
*Vrystaatia aloeicola*	CBS 135107	KF251781	–	KF251278	–
*Wingfieldomyces cyperi*	CBS 141450	KX228337	–	KX228286	MK540163
*Wojnowiciella eucalypti*	CPC 25024	KR476774	–	KR476741	LT990617
*W. kunmingensis*	KUMCC 18-0159	MK356354	MK356368	MK356380	MK359071
*Xenophoma puncteliae*	CBS 128022	JQ238619	–	–	KP170686
*Xenoseptoria neosaccardoi*	CBS 120.43	KF251783	–	KF251280	KF253227
	CBS 128665	KF251784	–	KF251281	KF253228
*Yunnanensis chromolaenae*	MFLUCC 17-1486	MN994573	MN994619	MN994596	MN998177
	MFLUCC 17-1487	MN994574	MN994620	MN994597	MN998178
*Yunnanensis phragmitis*	MFLUCC 17-0315	MF684863	MF684867	MF684862	MF683624
	MFLUCC 17-1361	MF684865	MF684864	MF684869	–

## Results

### Phylogenetic analysis

The combined dataset of ITS, LSU, SSU and *tef1*-α sequences comprised 3423 characters, of which 2418 characters are constant, 697 characters are parsimony-informative, while 308 variable characters are parsimony-uninformative in the maximum parsimony (MP) analysis (TL = 6364, CI = 0.250, RI = 0.657, RC = 0.164, HI = 0.750). The RAxML analysis of the combined dataset yielded a best scoring tree (Figure 1) with a final ML optimization likelihood value of – 34492.801018. The matrix had 1331 distinct alignment patterns, with 37.25% of undetermined characters or gaps. Estimated base frequencies are; A = 0.247120, C = 0.228182, G = 0.268238, T = 0.256459; substitution rates AC = 1.250439, AG = 3.526348, AT = 2.517351, CG = 0.798250, CT = 6.907432, GT = 1.000; proportion of invariable sites I = 0.596400; gamma distribution shape parameter α = 0.492378. All analyses (ML, MP and BI) gave similar results and are in agreement with previous studies based on multi-gene analyses (Hyde et al. 2019, 2020; Phookamsak et al. 2019). Phylogenetic analyses of the combined data matrix resulted in well-resolved clades, many of which had considerably high statistical support (Figure 1). Bootstrap support values for maximum likelihood, maximum parsimony ≥70%, and Bayesian posterior probabilities (BYPP) ≥0.95 are given above each branch in that order (Figure 1). Phylogenetic position and statistical support are noted in the taxonomy section.

**Figure 1.**
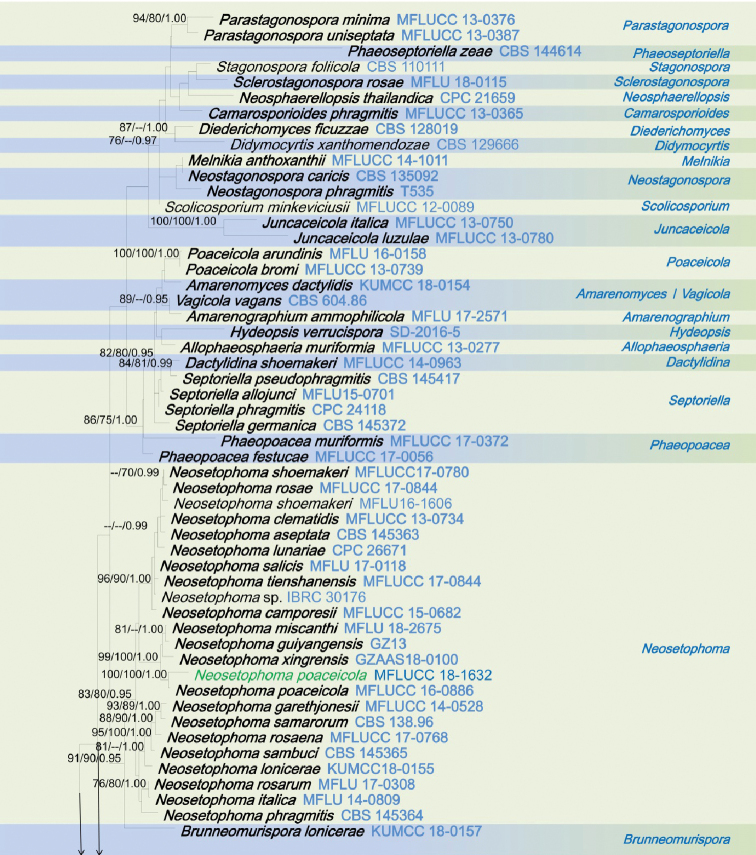
RAxML tree inferred from combined dataset of ITS, LSU, SSU and *tef1*-α partial sequences of 168 strains of Phaeosphaeriaceae. Bootstrap support values for maximum likelihood (ML), maximum parsimony (MP) values ≥70%, and Bayesian posterior probabilities (BYPP) ≥0.95 are given above each branch respectively. The new species are highlighted in red, and the new record in green. Ex-type strains are in bold. The tree is rooted by *Leptosphaeria doliolum* (CBS 505.75) and *Paraleptosphaeria dryadis* (CBS 643.86).

**Figure 1.**
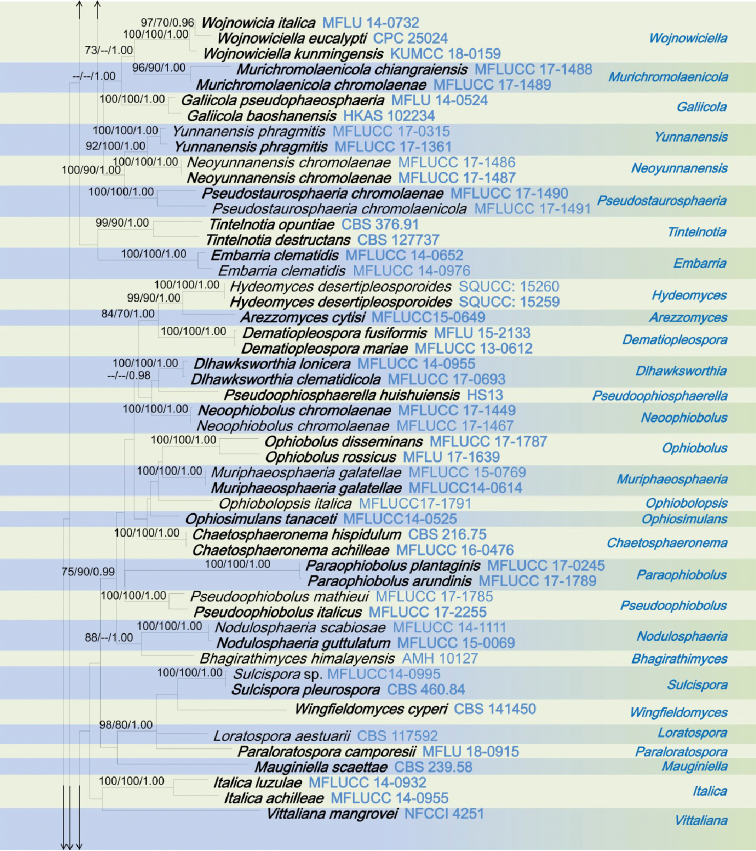
Continued.

**Figure 1.**
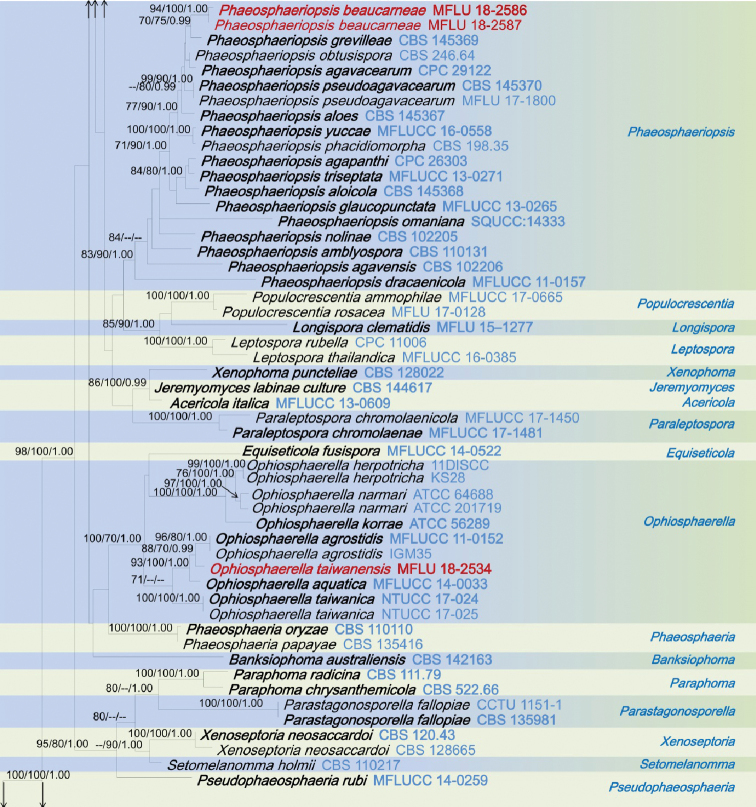
Continued.

**Figure 1.**
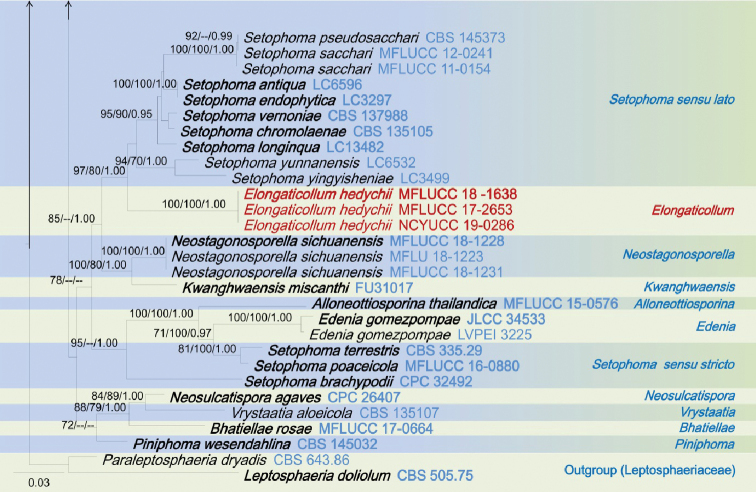
Continued.

### Taxonomy

#### Elongaticollum

Taxon classificationFungiPleosporalesPhaeosphaeriaceae

Tennakoon, C.H. Kuo & K.D. Hyde gen. nov.

099B265A-B2F3-55FD-85FE-20DDD861F112

Index Fungorum number: IF 557486

Facesoffungi number: FoF07849

##### Etymology.

Refers to the fact that the pycnidia have elongated necks.

##### Diagnosis.

*Saprobic* on dead leaves of *Hedychium coronarium* J. Koenig. **Sexual morph**: Undetermined. **Asexual morph**: Coelomycetous. *Conidiomata* pycnidial, solitary, superficial, dark brown to black, obpyriform, papillate. *Neck* elongate, dark brown, usually straight, but sometimes slightly curved. *Conidiomatal wall* composed of 4–5 layers of light brown cells, arranged in *textura angularis*. *Conidiophores* reduced to conidiogenous cells. *Conidiogenous cells* hyaline, aseptate, smooth, ampulliform, arising from the inner cell wall of the apex. *Conidia* oval to oblong, smooth and thin-walled, hyaline, aseptate, with 1–2-minute guttules.

##### Type species.

*Elongaticollum hedychii* Tennakoon, C.H. Kuo & K.D. Hyde.

#### Elongaticollum hedychii

Taxon classificationFungiPleosporalesPhaeosphaeriaceae

Tennakoon, C.H. Kuo & K.D. Hyde sp. nov.

934058B4-73A6-54E5-AD90-1F316FFB339C

Index Fungorum number: IF 557487

Facesoffungi number: FoF07850

Figure 2

##### Etymology.

Name reflects the host *Hedychium coronarium* J. Koenig, from which the holotype was collected.

##### Holotype.

MFLU 18-2542.

##### Diagnosis.

*Saprobic* on dead leaves of *Hedychium coronarium* J. Koenig. **Sexual morph**: Undetermined. **Asexual morph**: Coelomycetous. *Conidiomata* 120–140 µm high, 60–70 µm diam., pycnidial, solitary, scattered, superficial, visible as small black spots on host surface, dark brown to black, obpyriform, papillate. *Neck* up to 80–100 μm long, 20–30 µm diam., elongated, dark brown, usually straight, but sometimes slightly curved. *Conidiomatal wall* 10–20 µm wide, composed of 4–5 layers of light brown, thick-walled cells, arranged in *textura angularis*. *Conidiophores* reduced to conidiogenous cells. *Conidiogenous cells* 3–4 × 3–3.5 μm (*x̄* = 3.6 × 3.2 μm, n = 10), arising from the inner cell wall of the apex, hyaline, aseptate, smooth, ampulliform. *Conidia* 4–5 × 1.8–2.2 μm (*x̄* = 4.6 × 2.1 μm, n = 30), oval to oblong, smooth, thin-walled, hyaline, aseptate, with 1–2-minute guttules.

##### Culture characteristics.

Colonies on PDA reaching 30 mm diameter after 3 weeks at 20–25 °C, colonies medium sparse, circular, raised, surface slightly rough with entire edge, margin entire, colony from above: light brown to grey at the margin, dark brown at middle, dark brown to black at the center; reverse, light brown to yellowish at the margin, brown at middle, dark brown to black at the center; mycelium light brown to grey with tufts; not producing pigments in PDA.

##### Material examined.

Taiwan, Chiayi, Fanlu Township area, Dahu Forest, dead leaves of *Hedychium coronarium* J. Koenig (Zingiberaceae), 15 August 2018 (23°27.514'N, 120°36.302'E), D.S. Tennakoon, TLF031-A (MFLU 18-2542, ***holotype***), ex-type living culture (MFLUCC 18-1638 = NCYUCC 19-0163); *ibid*. 20 August 2018 (23°27.530'N, 120°36.314'E), TLF031-B (NCYU19-0139, ***paratype***), living culture (NCYUCC19-0286); *ibid*. 25 August 2018 (23°27.512'N, 120°36.301'E), TLF031-C (NCYU19-0140, ***paratype***), living culture (NCYUCC 19-0287).

##### Notes.

**Figure 2.**
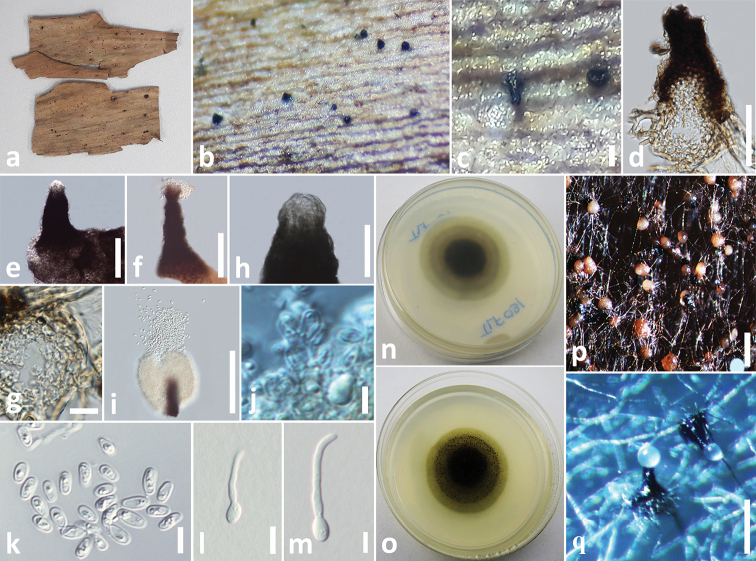
*Elongaticollum hedychii* (MFLU 18-2542, holotype) **a** specimen **b** appearance of conidiomata on host **c** close up of conidiomata on host **d** vertical section through conidioma **e, f** squash mount of conidioma **g** conidioma wall **h, i** elongated conidiomatal necks **j** conidiogenous cells **k** conidia **l, m** germinated conidia **n** colony from below **o** colony from above **p, q** pycnidia formed on PDA. Scale bars: 100 µm (**c**), 50 µm (**d–h**), 10 µm (**g**), 30 µm (**i**), 3 µm (**j–m**), 100 µm (**p, q**).

The genus *Elongaticollum* differs from other asexual morphs in Phaeosphaeriaceae in dark brown to black, superficial, obpyriform, pycnidial conidiomata with distinct elongate necks (80–100 μm) and a globose base and oval to oblong, hyaline, aseptate conidia (Figure 2). Multi-gene phylogenetic analyses (LSU, SSU, ITS, *tef1*-α), show *Elongaticollum* strains constitute a highly supported independent lineage nested between *Setophomasensu lato* and *Neostagonosporella* (97% ML, 80% MP, 1.00 BYPP, Figure 1). However, the asexual morph of *Setophoma* can be distinguished from *Elongaticollum* in having setose conidiomata without elongate necks and oblong to ellipsoidal conidia, whereas, *Elongaticollum* have conidiomata with distinct elongate necks and lacking setae and oval to oblong conidia (De Gruyter et al. 2010; Phookamsak et al. 2014). Despite some *Setophoma* species not having setae (i.e. *S. antiqua*, *S. endophytica*, and *S. yunnanensis*) (Liu et al. 2019), *Elongaticollum* species can be distinguished by its superficial conidiomata with elongate necks.

The asexual morph of *Neostagonosporella* differs from *Elongaticollum* in having multiloculate conidiomata without distinct elongate necks and two types of conidia (macroconidia: subcylindrical to cylindrical, transversely multi-septate, hyaline and microconidia oval, ellipsoidal or long ellipsoidal, aseptate, hyaline), whereas *Elongaticollum* has uni-loculate conidiomata with distinct elongate necks and oval to oblong conidia (Figure 2, Yang et al. 2019).

Phylogenetic investigations herein provide insights into the taxonomy of *Setophoma* as well (Figure 1). Two major clades of *Setophoma* are recovered (*Setophomasensu stricto* and *Setophomasensu lato.* The *Setophomasensu stricto* clade includes *S. brachypodii*, *S. poaceicola* and *S. terrestris* (type species). *Setophomasensu lato* comprises *S. antiqua*, *S. chromolaenae*, *S. endophytica*, *S. pseudosacchari*, *S. sacchari*, *S. vernoniae*, *S. yingyisheniae* and *S. yunnanensis* (Figure 1). *Elongaticollum*, differs from *Setophomasensu lato* in having distinct superficial, obpyriform, pycnidial conidiomata with a globose base and distinct elongated necks (Figure 2, Liu et al. 2019). Further work is needed to resolve relationships between *Setophomasensu stricto* and *Setophomasensu lato.*

#### Ophiosphaerella

Taxon classificationFungiPleosporalesPhaeosphaeriaceae

Speg., Anal. Mus. nac. B. Aires, Ser. 3 12: 401 (1909)

8624C0BF-7DAC-5E5D-9E1F-4F266473DD1C

##### Notes.

*Ophiosphaerella* was introduced by Spegazzini (1909) to accommodate *O. graminicola* Speg. as the type species. The species of this genus are characterized by papillate ascomata bearing fissitunicate, cylindrical asci frequently narrower near the base, with a short furcate pedicel and filamentous, pale brown, multi-septate ascospores without swollen cells or separating into part spores. Barr (1987) placed *Ophiosphaerella* in Phaeosphaeriaceae and this was confirmed by Zhang et al. (2009, 2012) and Hyde et al. (2013) based on molecular phylogeny. Most *Ophiosphaerella* species are often found as pathogens or saprobes worldwide on Poaceae and Cyperaceae (Câmara et al. 2000). Currently, twelve *Ophiosphaerella* species are listed in Index Fungorum (2020). In this study, we introduce *Ophiosphaerella taiwanensis* from *Agave tequilana* F.A.C. Weber (Asparagaceae) as a new species.

#### Ophiosphaerella taiwanensis

Taxon classificationFungiPleosporalesPhaeosphaeriaceae

Tennakoon, C.H. Kuo & K.D. Hyde sp. nov.

4D2BB74E-F917-5ACB-B0D0-16979236E2FF

Index Fungorum number: IF 557488

Facesoffungi number: FoF07851

Figure 3

##### Etymology.

Named after Taiwan, where this fungus was collected.

**Figure 3.**
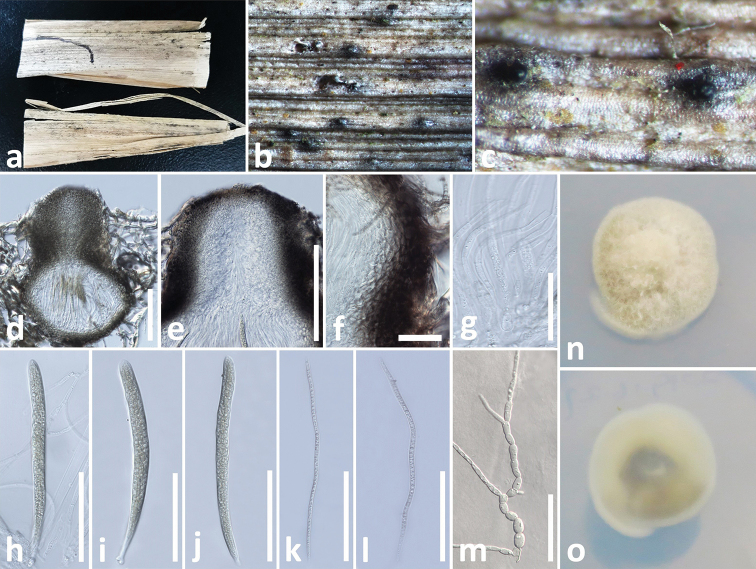
*Ophiosphaerella taiwanensis* (MFLU 18-2534, holotype) **a, b** appearance of ascomata on host **c** close-up of ascomata **d** vertical section through ascoma **e** apex of ascoma **f** peridium **g** pseudoparaphyses **h–j** asci **k, l** ascospores **m** germinated ascospore in PDA**n** colony from above **o** colony from below. Scale bars: 100 µm (**d, e**), 15 µm (**f**), 50 µm (**g–m**).

##### Holotype.

MFLU 18-2534.

##### Diagnosis.

*Saprobic* on dead leaf of *Agave tequilana* F.A.C. Weber (Asparagaceae). **Sexual morph**: *Ascomata* 270–310 μm high, 220–260 μm diam., solitary, scattered, immersed to slightly erumpent through host tissue with papilla, visible as raised, small black dots in host surface, globose to subglobose, uniloculate, glabrous, dark brown to black, ostiole central, periphysate. *Peridium* 20–25 μm wide, thick-walled, of equal thickness, composed of 6–7 layers of small, flattened, brown to dark brown pseudoparenchymatous cells, hyaline towards the inside, arranged in a *textura angularis*, fusing and indistinguishable from the host tissues. *Hamathecium* of 1.5–2.5 µm wide, cellular, septate, rarely branching, pseudoparaphyses, anastomosing mostly above the asci and embedded in a mucilaginous matrix. *Asci* 115–140 × 8.5–10 μm (*x̄* = 121.6 × 9.2 μm, n = 20), 8-spored, bitunicate, fissitunicate, cylindrical to cylindric-clavate, short pedicellate, apically rounded, with a well-developed ocular chamber. *Ascospores* 110–132 × 2.2–2.7 μm (*x̄* = 117.2 × 2.4 μm, n = 20), fasciculate, parallel, scolecosporous, filiform, 12–13-septate, narrowing towards ends, pale brown to brown, smooth-walled. **Asexual morph**: Undetermined.

##### Culture characteristics.

Colonies on PDA reaching 25 mm diameter after 3 weeks at 20–25 °C, colonies medium sparse, circular, raised, surface slightly rough with entire edge, margin well-defined, colony from above: gray to light brown at the margin, gray to cream at the center; reverse, gray to light brown at the margin, dark brown to black at the center; mycelium whitish gray with tufting; not producing pigments in PDA.

##### Material examined.

Taiwan, Chiayi, Fanlu Township area, Dahu Forest, dead leaf of *Agave tequilana* F.A.C. Weber (Asparagaceae), 15 August 2018 (23°27.520'N, 120°36.310'E), D.S. Tennakoon, TLF016 (MFLU 18-2534, ***holotype***); *ibid*. (NCYU19-0131, ***isotype***), ex-type living culture, NCYUCC 19-0152.

##### Notes.

The scolecosporous specimen was collected from dead leaves of *Agave tequilana* (Asparagaceae) in Taiwan. The multi-gene phylogenetic analysis (Figure 1) shows our strain (*Ophiosphaerella taiwanensis*, NCYUCC 19-0152), cluster with other *Ophiosphaerella* species, in particular with close affinity to *Ophiosphaerella agrostidis* with high bootstrap support (88% ML, 70% MP, 0.99 BYPP, Figure 1). Morphological characters of our collection (NCYUCC 19-0152) differ from *Ophiosphaerella agrostidis* in having periphyses in the ostiole, 12–13 septate ascospores and host occurrence (Asparagaceae). *Ophiosphaerella agrostidis* was introduced by Câmara et al. (2000) on *Agrostis palustris* (Poaceae), and is lacking periphyses, comprises 15-septate ascospores (Phookamsak et al. 2014). A comparison of the 619 nucleotides across the *tef1*-α gene region of *Ophiosphaerella taiwanensis* and *O. agrostidis* (MFLUCC 11-0152) reveals 17 base pair differences (2.74%).

#### Phaeosphaeriopsis

Taxon classificationFungiPleosporalesPhaeosphaeriaceae

M.P.S. Câmara, M.E. Palm & A.W. Ramaley, Mycol. Res. 107(5): 519 (2003)

06CCEE9D-81EC-5D0B-967E-99E7810387E3

##### Notes.

The genus *Phaeosphaeriopsis* was introduced by Câmara et al. (2003) to accommodate *Paraphaeosphaeria*-like taxa, viz. *P. agavensis* A.W. Ramaley, M.E. Palm & M.E. Barr, *P. glaucopunctata* (Grev.) Shoemaker & C.E. Babc., *P. nolinae* A.W. Ramaley, *P. obtusispora* (Speg.) O.E. Erikss, *Phaeosphaeriopsis amblyspora* A. W. Ramaley and *Phaeosphaeriopsis amblyspora* A. W. Ramaley. The genus is typified by *P. glaucopunctata* and characterized by having immersed, sub-epidermal, globose to subglobose to pyriform ascomata, cylindric asci and septate, punctate or verrucose ascospores (Câmara et al. 2003; Phookamsak et al. 2014; Thambugala et al. 2014; Tibpromma et al. 2017). Currently, 17 *Phaeosphaeriopsis* species are accepted in Index Fungorum (2020). In this paper, *Phaeosphaeriopsis beaucarneae* is introduced from *Beaucarnea recurvata* (Asparagaceae) as a new species and the sexual/asexual morph connection between strains isolated from the natural habitat was established based on molecular sequence data.

#### Phaeosphaeriopsis beaucarneae

Taxon classificationFungiPleosporalesPhaeosphaeriaceae

Tennakoon, C.H. Kuo & K.D. Hyde sp. nov.

B91A27ED-8901-57B1-87F7-C3FF701314F0

Index Fungorum number: IF 557489

Facesoffungi number: FoF07852

Figures 4 , 5

##### Etymology.

Name reflects the host *Beaucarnea recurvata* Lem., from which the holotype was collected.

**Figure 4.**
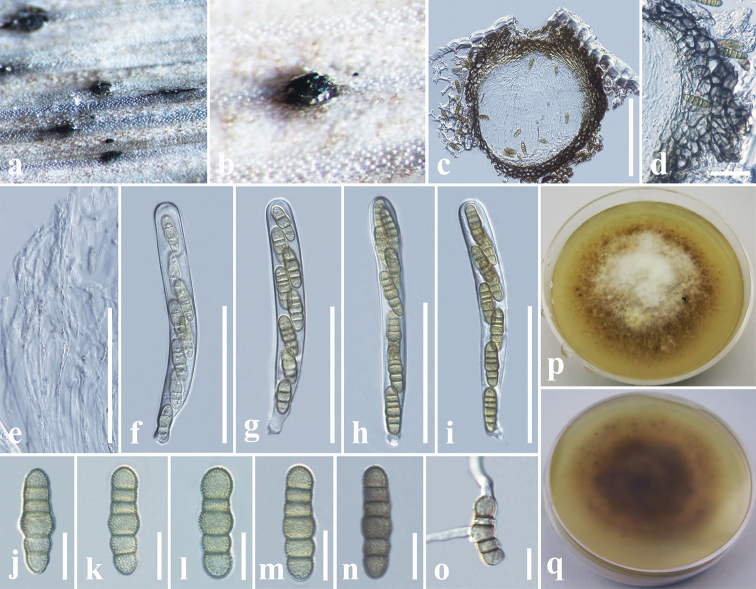
*Phaeosphaeriopsis beaucarneae* (MFLU 18-2586, holotype) **a** appearance of ascomata on host **b** close up of ascoma **c** vertical section through ascoma **d** peridium **e** pseudoparaphyses **f–i** asci **j–n** ascospores **o** germinated ascospore in PDA**p** colony from above **q** colony from below. Scale bars: 100 µm (**c**), 15 µm (**d**), 50 µm (**e–i**), 10 µm (**j–o**).

**Figure 5.**
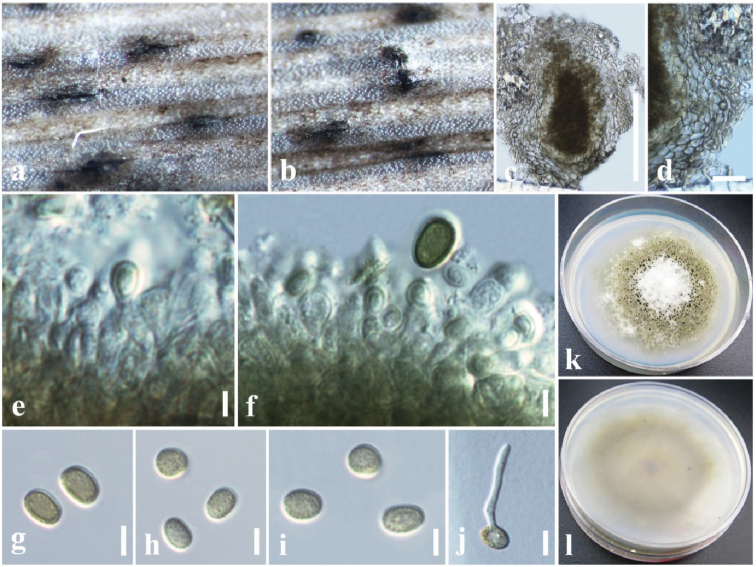
*Phaeosphaeriopsis beaucarneae* (MFLU 18-2586, paratype) **a** appearance of conidiomata on host **b** close up of conidiomata **c** vertical section through conidioma **d** conidiomatal wall **e, f** conidiogenous cells and developing conidia **g–i** conidia **j** germinated conidium in PDA**k** colony from above **l** colony from below. Scale bars: 100 µm (**c**), 20 µm (**d**), 3 µm (**e, f**), 5 µm (**g–j**).

##### Holotype.

MFLU 18-2586.

##### Diagnosis.

*Saprobic* on dead leaf of *Beaucarnea recurvata* Lem. (Asparagaceae). **Sexual morph**: *Ascomata* 160–200 μm high, 220–250 μm diam., scattered, solitary, gregarious, coriaceous, immersed to semi-immersed, slightly raised, erumpent, visible as black spots on host surface, uniloculate, dark brown to black, globose to subglobose, ostiolate. *Ostiole* central, papillate. *Peridium* 20–30 μm wide, thick-walled, of equal thickness, composed of 4–5 layers of dark brown to brown, thick-walled, pseudoparenchymatous cells of *textura angularis*. *Hamathecium* of 1.5–2.5 µm wide, cellular, septate, rarely branching, pseudoparaphyses, anastomosing mostly above the asci and embedded in a mucilaginous matrix. *Asci* 80–90 × 9–10 µm (*x̄* = 86.5 × 9.6 µm, n = 25), 8-spored, bitunicate, fissitunicate, cylindrical to cylindric-clavate, short pedicellate, apically rounded, with a well-developed ocular chamber. *Ascospores* 20–25 × 5.5–7 μm (*x̄* = 22.6 × 6.2 μm, n = 20), overlapping 1–2-seriate, oblong to cylindrical, yellowish to light brown, slightly narrowing towards the end cells, mostly 5-septate, constricted at the septa, enlarged at the 4^th^ cell from above, verruculose, straight to curved, lacking a mucilaginous sheath. **Asexual morph**: *Conidiomata* 180–200 µm high, 140–160 µm diam., pycnidial, solitary, immersed to erumpent, small black spots on host surface, globose to subglobose with centrally placed ostiole. *Conidiomatal wall* 28–34 µm wide, composed of 6–7 layers of dark brown cells, arranged in *textura angularis*. *Conidiophores* reduced to conidiogenous cells. *Conidiogenous cells* 3–4 × 2.6–3.1 μm, holoblastic, phialidic, single, discrete, sometimes integrated, ampulliform or cylindric-clavate, hyaline, arising from basal stratum. *Conidia* 6.8–7.4 × 3–4 μm (*x̄* = 7.1 × 3.4 μm, n = 30), 1-celled, globose to subglobose, initially hyaline, becoming brown to dark brown, aseptate, rough-walled.

##### Culture characteristics.

Colonies on PDA reaching 27 mm diameter after 3 weeks at 20–25 °C, colonies medium sparse, circular, raised, surface slightly rough with entire edge, margin irregular, colony from above: light brown at the margin, white to cream at the center; reverse, yellow to light brown at the margin, light brown to brown at the center; mycelium white to cream with tufting; not producing pigments in PDA.

##### Material examined.

Taiwan, Chiayi, Fanlu Township area, Dahu Forest, dead leaf of *Beaucarnea recurvata* Lem. (Asparagaceae), 21 July 2018 (23°27.514'N, 120°36.302'E), D.S. Tennakoon, SV027 (MFLU 18-2586, ***holotype***); *ibi.* (NCYU19-0184, ***isotype***), ex-type living culture, NCYUCC 19-0106; *ibid*., Dahu forest, dead leaf of *Beaucarnea recurvata* Lem. (Asparagaceae), 25 July 2018 (23°26.534'N, 120°36.220'E), D.S. Tennakoon, SV028 (MFLU 18-2587, ***paratype***); living culture, NCYUCC 19-0107.

##### Notes.

*Phaeosphaeriopsis beaucarneae* is similar to other *Phaeosphaeriopsis* species in having scattered, semi-immersed to erumpent, globose to subglobose, ostiolate ascomata and cylindrical to clavate asci and light brown, verrucose ascospores (Phookamsak et al. 2014; Thambugala et al. 2014; Hyde et al. 2020). According to the present multi-gene phylogenetic analyses (Figure 1), *Phaeosphaeriopsis beaucarneae* is grouped with other *Phaeosphaeriopsis* species, in particularly closely to *P. grevilleae* (CBS 145369) with high statistical support (70% ML, 75% MP, 0.99 BYPP, Figure 1). The asexual morph of *P. grevilleae* was isolated from leaves of *Grevillea* sp. (Proteaceae) and introduced by Marin-Felix et al. (2019). *Phaeosphaeriopsis beaucarneae* differs from *P. grevilleae* in having larger conidia (6.8–7.4 × 3–4 μm), whereas *P. grevilleae* has comparatively smaller conidia (5 × 3.5 μm). A comparison of the 516 nucleotides across the ITS (+5.8S rDNA) gene region of *Phaeosphaeriopsis beaucarneae* and *P. grevilleae* (CBS 145369) revealed 16 base pair differences (3.10%). In addition, we compared our new taxon with *P. grevilleae* based on base pair differences in the *tef1*-α gene region. We found a total of 19 base pair differences (3.06%) across 619 nucleotides.

Recent studies have revealed that *Phaeosphaeriopsis* is a species rich genus and numerous *Phaeosphaeriopsis* species have been described during the last few years (Thambugala et al. 2014; Tibpromma et al. 2017; Marin-Felix et al. 2019; Al-Jaradi et al. 2020; Hyde et al. 2020). With this study, the number of *Phaeosphaeriopsis* species increases to 18.

#### Neosetophoma

Taxon classificationFungiPleosporalesPhaeosphaeriaceae

Gruyter, Aveskamp & Verkley, Mycologia 102(5): 1075 (2010)

A4D85E50-AC97-57DC-B533-2418745AD589

##### Notes.

*Neosetophoma* was introduced by de Gruyter et al. (2010), typified by *N. samararum* (Desm.) Gruyter, Aveskamp. & Verkley. Species of *Neosetophoma* are characterized by globose to irregular conidiomata, with papillate ostioles, and yellowish conidia that are attenuate at one end (De Gruyter et al. 2010; Liu et al. 2015). Tibpromma et al. (2017) introduced *Neosetophoma garethjonesii* Tibpromma, E.B.G. Jones & K.D. Hyde as the first report of the sexual morph of *Neosetophoma*. *Neosetophoma* species have a diverse distribution as saprobes, endophytes, plant pathogens and soil fungi (Phookamsak et al. 2014; Hernandez-Restrepo et al. 2016; Karunarathna et al. 2017; Tibpromma et al. 2017; Wanasinghe et al. 2018). Currently, 19 *Neosetophoma* species are accepted in Index Fungorum (2020). In this study, we found *Neosetophoma poaceicola* Goonas., Thambug. & K.D. Hyde from dead leaves of *Musa acuminata* Colla in Taiwan. This is the first *Neosetophoma* species recorded from the plant family Musaceae.

#### Neosetophoma poaceicola

Taxon classificationFungiPleosporalesPhaeosphaeriaceae

Goonas., Thambug. & K.D. Hyde. Mycosphere 8: 742 (2017)

4C1AA9B6-EA48-5A22-89E6-0AF230FD2C5A

Index Fungorum number: IF552974

Facesoffungi number: FoF00262

Figure 6

##### Diagnosis.

*Saprobic* on dead leaf petioles of *Musa acuminata* Colla (Musaceae). **Sexual morph**: *Ascomata* 70–100 μm high, 90–130 μm diam., solitary, gregarious, coriaceous, immersed to semi-immersed, slightly raised, visible as black spots on host surface, uni-loculate, dark brown to black, globose to ovoid. *Peridium* 15–20 μm wide, thick-walled, of equal thickness, composed of several layers of dark brown to brown, pseudoparenchymatous cells of *textura angularis*. *Hamathecium* of 1–2 µm wide, cellular, rarely branching, pseudoparaphyses, anastomosing mostly above the asci and embedded in a mucilaginous matrix. *Asci* 60–80 × 7–8 μm (*x̄* = 70.6 × 7.6 μm, n = 30), 8-spored, bitunicate, fissitunicate, cylindric-clavate with a short, rounded pedicel, apically rounded. *Ascospores* 20–30 × 3–4 μm (*x̄* = 25.5 × 3.7 μm, n = 40), overlapping 1–2-seriate, hyaline, fusiform, with acute ends, 1-septate, 3–4 eu-septate, cell near the septum slightly larger, slightly constricted at the septum, straight to curved, smooth-walled, guttulate. **Asexual morph**: Undetermined.

##### Culture characteristics.

Colonies on PDA reaching 30 mm diameter after 3 weeks at 20–25 °C, colonies medium sparse, circular, flat, surface slightly rough with entire edge, margin well-defined, colony from above: yellow to light brown at the margin, brown at the center; reverse, yellow to light brown at the margin, dark brown at the center; mycelium light brown to whitish grey with tufting; not producing pigments in PDA.

##### Material examined.

Taiwan, Chiayi, Fanlu Township area, Dahu Forest, dead leaf petiole of *Musa acuminata* Colla (Musaceae), 21 July 2018 (23°27.530'N, 120°36.340'E), D.S. Tennakoon, SV049 (MFLU 18-2597, **new host record**), living culture, MFLUCC 18-1632, NCYUCC 19-0119.

##### Notes.

As morphological characters (immersed to semi-immersed ascomata, cylindric-clavate, apically rounded asci with short rounded pedicel and hyaline, fusiform, 1-septate ascospores) largely overlap with those of *Neosetophoma poaceicola* (MFLUCC 16–0886), we report our collection (MFLUCC 18-1632) as a new host record of *N. poaceicola* from dead leaves of *Musa acuminata* (Musaceae) in Taiwan. Combined multi-gene (LSU, SSU, ITS and *tef1*-α) based phylogenies also showed that our collection clustered with *Neosetophoma poaceicola* (MFLUCC 16-0886), with high bootstrap support (100% ML, 100% MP, 1.00 BYPP, Figure 1). *Neosetophoma poaceicola* was introduced by Thambugala et al. (2017) from dead leaves of grass species in Thailand. However, our collection slightly differs from *Neosetophoma poaceicola* (MFLUCC 16-0886) in having comparatively slightly larger ascospores (20–30 × 3–4 μm, versus 18.5–22.5 × 3.5–5 μm).

*Neosetophoma* species have been recorded from various host families, viz. Brassicaceae, Caprifoliaceae, Iridaceae, Malvaceae, Ranunculaceae, Salicaceae, but most are reported from Poaceae (Phookamsak et al. 2014; Karunarathna et al. 2017; Tibpromma et al. 2017, Wanasinghe et al. 2018; Marin-Felix et al. 2019). Interestingly, this is the first *Neosetophoma* species record (MFLU 18-2597) from the plant family Musaceae.

**Figure 6.**
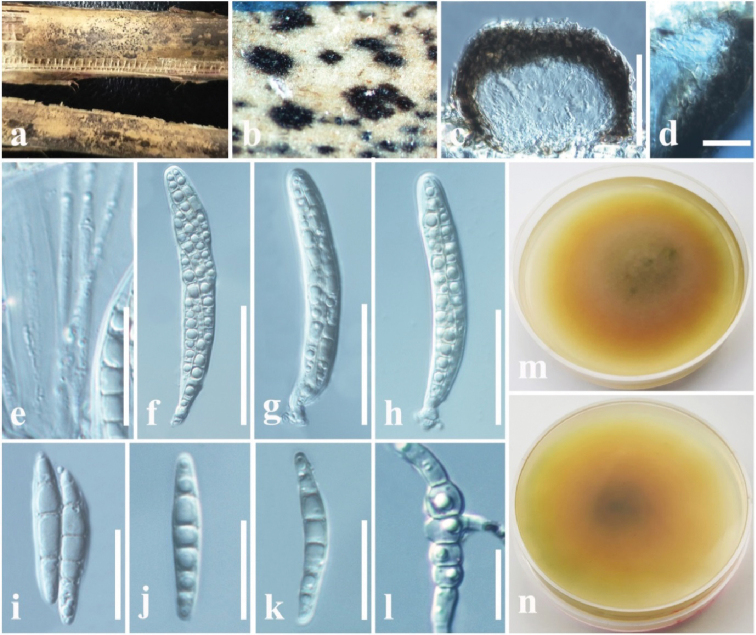
*Neosetophoma poaceicola* (MFLU 18–2597, new host record) **a** appearance of ascomata on host **b** close up of ascomata **c** vertical section through ascoma **d** peridium **e** pseudoparaphyses **f–h** asci **i–k** ascospores **l** germinated ascospore in PDA**m** colony from above **n** colony from below. Scale bars: 50 µm (**c**), 20 µm (**d**), 30 µm (**e–h**), 15 µm (**i–l**).

## Discussion

The taxonomy of Phaeosphaeriaceae has been subjected to several changes in recent years. Traditionally, morphology-based identification was the main means for identifying Phaeosphaeriaceae species (Barr 1979, 1992; Tomilin 1993). However, species identification has been revolutionized by the application of molecular based approaches incorporating DNA sequence data in Phaeosphaeriaceae (Phookamsak et al. 2014, 2017; Tennakoon et al. 2016; Wanasinghe et al. 2018; Bakhshi et al. 2019; Chethana et al. 2020; Hyde et al. 2020). Phaeosphaeriaceae species are adapted to a wide range of ecological environments and are present in soils, fresh and marine habitats and cause infections in humans (Yuan 1994; Phookamsak et al. 2014, 2017; Ahmed et al. 2017; Maharachchikumbura et al. 2019; Valenzuela-Lopez et al. 2019). Members of the Phaeosphaeriaceae have also been recorded from both temperate and tropical countries (i.e. Austria, Belgium, Bulgaria, Canada, China, Germany, Italy, Japan, Norway, Poland, Thailand, Sweden, Switzerland) and from different host families (i. e. Acoraceae, Arecaceae, Cyperaceae, Asparagaceae, Brassicaceae, Fabaceae, Poaceae, Marantaceae) (Shoemaker and Babcock 1989; Phookamsak et al. 2014, 2019; Wanasinghe et al. 2018; Maharachchikumbura et al. 2019; Farr and Rossman 2020). Due to their cosmopolitan distribution, in the last few years, many researchers have paid significant attention to the Phaeosphaeriaceae (Phookamsak et al. 2014, 2019; Tennakoon et al. 2016; Wanasinghe et al. 2018; Bakhshi et al. 2019; Hyde et al. 2020).

The fungi that decay leaf litter are highly diverse and may be host-specific (Parungao et al. 2002). Several studies have examined the succession of leaf degrading communities and found unique sets of species on different types of litter (Promputtha et al. 2002, 2017; Duong et al. 2008). Additional ecological studies are therefore needed to establish whether these fungi are generalists or specialists. This study provides evidence to indicate the fungal diversity in leaf litter, even within a single family, Phaeosphaeriaceae. Additional work is necessary to identify if the newly described species are host specific.

## Supplementary Material

XML Treatment for Elongaticollum

XML Treatment for Elongaticollum hedychii

XML Treatment for Ophiosphaerella

XML Treatment for Ophiosphaerella taiwanensis

XML Treatment for Phaeosphaeriopsis

XML Treatment for Phaeosphaeriopsis beaucarneae

XML Treatment for Neosetophoma

XML Treatment for Neosetophoma poaceicola
